# Bimonthly Administered Long-Acting Cabotegravir and Rilpivirine Are Highly Effective and Well-Tolerated in People With Human Immunodeficiency Virus Above 65 Years

**DOI:** 10.1093/ofid/ofaf817

**Published:** 2026-01-06

**Authors:** Andrea Calcagno, Caterina Candela, Agostino Riva, Stefano Calza, Benedetta Fioretti, Samuele Gardini, Jovana Milic, Benedetto Maurizio Celesia, Giancarlo Orofino, Andrea De Vito, Giuseppe Vittorio De Socio, Maria Vittoria Cossu, Federica Barrera, Maria Mazzitelli, Silvia Nozza, Giovanni Guaraldi, Emanuele Focà, for the GEPPO Study Group, Stefania Arsuffi, Domenico Azzolino, Marta Baroni, Giuseppe Bellelli, Luca Bonaffini, Paolo Bonfanti, Andrea Calcagno, Stefano Calza, Annamaria Cattelan, Benedetto Maurizio Celesia, Alessandra Coin, Giuseppe De Socio, Giovanni Di Perri, Micol Ferrara, Benedetta Fioretti, Emanuele Focà, Giovanni Guaraldi, Francesca Italiani, Alessandro Lazzaro, Tiziano Lucchi, Maria Grazia Maddalone, Giordano Madeddu, Alessandra Marengoni, Claudio Mastroianni, Jovana Milić, Chiara Mussi, Silvia Nozza, Giancarlo Orofino, Lavinia Patetta, Andrea Piazzoli, Stefania Piconi, Paola Pignata, Silvia Pontiggia, Agostino Riva, Anna Spolti

**Affiliations:** Department of Translational Medicine, University of Eastern Piedmont, Novara, Italy; Infectious Disease Unit, Università Vita Salute San Raffaele, Milano, Italy; Department of Infectious Diseases, University of Milan, ASST Fatebenefratelli Sacco University Hospital, Milan, Italy; Unit of Biostatistics and Bioinformatics, Department of Molecular and Translational Medicine, University of Brescia, Brescia, Italy; Department of Infectious and Tropical Diseases, University of Brescia and ASST Spedali Civili Hospital, Brescia, Italy; Infectious and Tropical Diseases Unit, Padua University Hospital, Padua, Italy; Department of Surgical, Medical, Dental and Morphological Sciences, University of Modena and Reggio Emilia, Modena, Italy; Division of Infectious Diseases, Department of Clinical and Molecular Biomedicine, University of Catania, Catania, Italy; Unit of Infectious Diseases, “Divisione A”, ASL “Città di Torino”, Torino, Italy; Unit of Infectious Diseases, Department of Medicine, Surgery and Pharmacy, University of Sassari, Sassari, Italy; Infectious Diseases Clinic, Department of Medicine and Surgery, Azienda Ospedaliera and University of Perugia, Santa Maria Hospital, Perugia, Italy; Department of Infectious Diseases, University of Milan, ASST Fatebenefratelli Sacco University Hospital, Milan, Italy; Unit of Infectious Diseases, Department of Medical Sciences, University of Turin, Turin, Italy; Infectious and Tropical Diseases Unit, Padua University Hospital, Padua, Italy; Dipartimento di Sicurezza e Bioetica - Sezione di Malattie Infettive, Università Cattolica del Sacro Cuore, Rome, Italy; Dipartimento di Scienze Mediche e Chirurgiche, Fondazione Policlinico Universitario Agostino Gemelli IRCCS, Rome, Italy; Infectious Disease Unit, Università Vita Salute San Raffaele, Milano, Italy; Department of Surgical, Medical, Dental and Morphological Sciences, University of Modena and Reggio Emilia, Modena, Italy; Department of Infectious and Tropical Diseases, University of Brescia and ASST Spedali Civili Hospital, Brescia, Italy

**Keywords:** elderly, hepatitis b reactivation, injectable, polypharmacy, safety

## Abstract

Older people with HIV (PWH) may benefit from long-acting cabotegravir/rilpivirine (LA-CAB/RPV), a population underrepresented in trials and observational cohorts. In the GEPPO cohort, 135 PWH >65 years of age received bimonthly LA-CAB/RPV. After 17.4 months, virological suppression (HIV-RNA <50 copies/mL) was maintained in all, while 15 participants discontinued them (11.1%, 10% in the first 12 months): 1 participant with isolated HbCAb at baseline showed a late HBV reactivation (19.4 months after starting). These findings support LA-CAB/RPV's efficacy and tolerability in older PWH.

The majority of people with HIV-1 (PWH) receiving combination antiretroviral treatment (cART) can control HIV replication and attain a good immunological status. The key challenge of contemporary clinical care is the prevention and management of comorbidities whose prevalence and impact are significantly enhanced in PWH [1]. A relatively recent field of HIV care is geriatric HIV medicine that focuses on older PWH (OPWH) since frailty, geriatric syndromes, and, ultimately, mortality seem to have a higher incidence in comparison to older people without HIV [2]. One of the objectives of geriatric HIV medicine is to assess cART in OPWH to identify drugs and combinations that, provided the expected high efficacy, may be associated with the least toxicity and potential to worsen aging-associated processes.

Long-acting injectable antiretroviral drugs have been recently introduced as cART, and they are in development for HIV treatment and prevention. Clinical trials and observation studies have reported that intramuscularly administered long-acting cabotegravir and rilpivirine (LA-CAB/RPV) are highly efficacious (with virological suppression maintained in >95% of individuals) and well-tolerated (with discontinuation rates between 5% and 10%, mostly due to injection site reactions) [3]. Yet, the uncommon virological failures are associated with a high risk of selecting major resistance-associated mutations (RAMs) [4]. Some risk factors for such failures have been identified (the pre-existing selection of RPV RAMs, the HIV A1/A6 genotype, and body mass index, BMI, ≥30 kg/m^2^) using multivariate models of pooled data from phase 3 randomized trials, while others are still being evaluated (low drug exposure and previous virological control) [5–8].

Data in OPWH are still limited since the average age in clinical trials and in most of the phase IV studies was below or around 50 years. A pharmacokinetic modeling study predicted CAB and RPV exposures higher than in young PWH and hypothesized a lower risk of suboptimal concentrations of both drugs [9]. Yet the efficacy, acceptability, and tolerability of LA-CAB/RPV need to be assessed in OPWH, given the high prevalence of multimorbidity, polypharmacy (PP), frailty, and sarcopenia (that may potentially affect the outcomes of intramuscularly administered drugs) that have been reported in this group of PWH.

The objective of this study was to evaluate the efficacy, durability, and safety of LA-CAB/RPV in OPWH as well as to identify the reasons for treatment discontinuation among those who stopped LA-CAB/RPV.

This was an observational retrospective analysis of OPWH (age ≥65 years) from the GEPPO cohort. All participants signed a written informed consent for cohort participation and for this specific study: the study protocol was approved by the coordinating institution (University of Brescia, CET Lombardia 3, ID 712_2018 and 720_SA_01.09.2023) and all participating centers.

For this analysis, we included all PWH in the GEPPO cohort who have received at least 1 dose of bimonthly intramuscularly administered LA-CAB + RPV between 2020 and 2024. Baseline (BL) was defined as the time when participants started LA-CAB + RPV. Duration of LA-CAB + RPV administration, reasons for discontinuation, and HIV-RNA/CD4+ T-cell count at the last available observation were recorded together with multimorbidity (defined as the presence of ≥3 comorbidities) and PP (defined as the use of ≥5 medications other than antiretroviral therapy). Isolated anti-core Hepatitis B Virus (HBV) was defined as the presence of antibodies against anti HBV core antigen (anti-HBc) with negative antibodies against HBV surface antigen (anti-HBs). The visits schedule as well as the management of viral blips/virological failures were according to clinical practice but all centers had a bimonthly follow-up in the first year and retested detectable viremias: loss of virological suppression was defined as 2 consecutive HIV-RNA >50 copies/mL. As for label, patients with RPV RAMs were not prescribed LA-CAB/RPV.

Data are shown as numbers (percentages) or medians (interquartile ranges or IQR): discontinuation rates were described with Kaplan–Meier curves and probabilities (with 95% CIs). Missing data are shown for each variable.

We included 135 participants with a median age of 67.8 (IQR: 65.0–71.0) years. Baseline characteristics are shown in Supplementary Table 1. Briefly, they were mostly male (85.9%) with a high CD4 cell count (607 cell/mm^3^, IQR: 479–929) and with undetectable HIV-RNA (97.7%). Multimorbidity (30.4%) and PP (64.4%) were common. The most common comorbidities were dyslipidemia (51.9%), arterial hypertension (31.9%), and osteopenia/osteoporosis (20%). Features associated with LA-CAB/RPV failure were uncommonly present (15% with body mass index or BMI ≥ 30 kg/m^2^, 6% A1/A6 genotype, and 3.8% pre-existing non-nucleoside reverse transcriptase inhibitor RAMs): only 3 participants (2.7%) showed 2 of these characteristics.

After a median follow-up of 17.4 months (CI 95% 1.0–76.0), 120 participants (88.9%) were still receiving LA-CAB + RPV; 8 (6%) participants had follow-up above 5 years since they had started LA-CAB + RPV in phase II/III clinical trials (Figure 1). No virological failure was observed, HIV-RNA remained <50 copies/mL in all PWH, and median CD4+ T-cell count at the last available observation was 660 (IQR 504–867) cells/mm^3^. Additionally, the 3 participants who had an HIV-RNA >50 copies/mL at BL (55, 234, and 945 copies/mL) achieved and maintained undetectable viral loads throughout follow-up. BMI at last observation was unchanged with a median differential value of 0 kg/m^2^ (IQR −0.6 − + 0.5).

**Figure 1.**
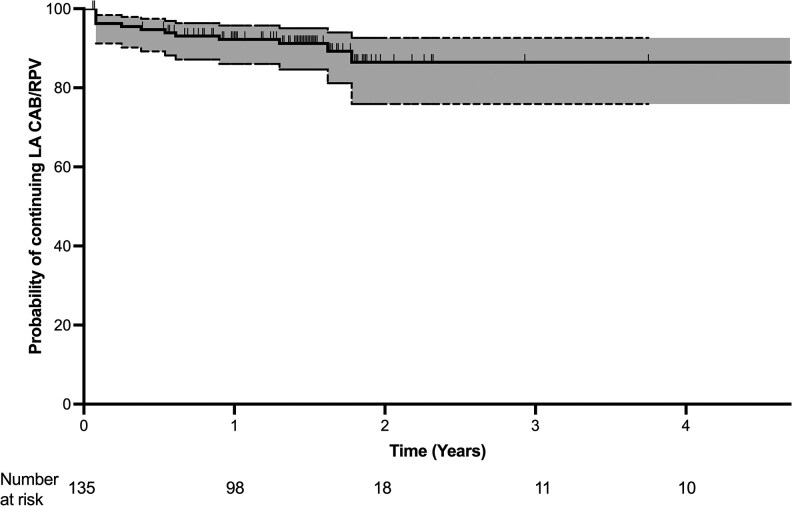
Kaplan–Meier curve showing the probability (and 95% CI) of continuing LA–CAB/RPV. The curve was truncated at 5 y of follow-up. LA-CAB/RPV, long-acting cabotegravir and rilpivirine.

The detailed reasons for discontinuation are shown in Supplementary Table 2. Among the 15 participants who interrupted LA-CAB/RPV, the main reasons were toxicity or intolerance [7], participants’ choice [6], or others. In the former group, injection site reactions, myalgia, and fever were the most common reasons. Of note, 1 participant with isolated HBc antibody positivity had a hepatic flare with aspartate aminotransferase (AST), alanine aminotransferase (ALT), and HBV DNA increase after 19.4 months of receiving LA-CAB/RPV. Finally, 1 participant with an undetectable viral load was discontinued after multidisciplinary discussion because of pre-existing non-nucleoside reverse transcriptase inhibitors (NNRTI) RAMs.

Data from randomized clinical trials and observational cohorts from different settings worldwide showed the efficacy and safety of LA-CAB/RPV [10]. Yet, most of the participants were either young or middle-aged PWH. Understanding the efficacy and safety can be relevant since OPWH have a higher risk of treatment-associated toxicity and may have sarcopenia that could impair intramuscularly administered drugs’ pharmacokinetics. An additional concern is that multimorbidity and PP—both highly prevalent—may reduce the patients’ preference and lead to additional or uncommon side effects. A subanalysis of the RELATIVITY study in PWH above 60 years was recently published: high efficacy (virological failure in 0.3% participants) and low discontinuation rates (7.8%, 1.6% adverse events) were reported [11].

Our data indicate that LA-CAB/RPV remains effective and acceptable in older adults with multimorbidity. Discontinuations were mainly patient-driven and not due to virological failure. After a median follow-up of 1.5 years, we observed no virological failure despite some of our participants having risk factors at BL, and 3 had a nonsuppressed viral load. The maintenance of virological control in 100% of our study participants is in line with global data suggesting that viral rebound is observed in <2% of people starting LA-CAB/RPV [3]. Besides, the phase III-derived risk factors have been challenged by several observational data, and, recently, we have off-label experiences in selected PWH with detectable viral load before switching [12].

LA-CAB/RPV treatment discontinuation has been reported in 10%–15% of PWH with longer follow-up in observational studies: 11.1% of our participants stopped LA-CAB/RPV (10% within 1 year) thus suggesting similar discontinuation rates [13].

The main reasons for treatment discontinuation were patients’ choice and injection site reactions, often associated with persistent or intense muscular pain. While sarcopenia could theoretically influence the perception of pain and injection tolerability, no direct measures of muscle mass strength or frailty were available in this cohort. Additionally, the need to attend the clinic every 2 months may represent a practical barrier for frail patients or those with mobility limitations. Conversely, PP itself did not appear to hinder access to long-acting therapy; consistent with previous studies, the decision to switch to an injectable regimen may be driven by the desire to reduce the perceived stigma associated with daily oral antiretroviral intake rather than by the actual number of pills taken. Patient-reported outcomes may help to better understand the factors influencing treatment decisions in OPWH.

An additional relevant issue is the occurrence, in 1 of our participants, of a hepatic flare due to HBV reactivation. This has been reported in PWH with isolated anti-HBc antibodies not receiving HBV-active antiretrovirals. Our participant had a mild hepatic flare with HBV reactivation 19.4 months after LA-CAB/RPV initiation, later than what was previously observed (usually 6–12 months): immune system changes and low reservoirs can potentially explain this difference. There is an ongoing discussion on how to manage PWH with past HBV coinfection [14].

These are preliminary results, and their generalizability may be limited by the small sample size, potential selection bias, heterogeneous follow-up and the restricted set of participants’ characteristics evaluated. Nonetheless, real-world data such as these are essential to inform the use of new therapeutic strategies in special populations, including OPWH.

In conclusion, LA-CAB/RPV was effective and well tolerated in PWH over 65 years: older age should not be a barrier *per se* to this treatment strategy in PWH.

## Supplementary Material

ofaf817_Supplementary_Data

## Supplementary Data

Supplementary materials are available at *Open Forum Infectious Diseases* online. Consisting of data provided by the authors to benefit the reader, the posted materials are not copyedited and are the sole responsibility of the authors, so questions or comments should be addressed to the corresponding author.
