# Supplementary material for: Bimonthly Administered Long-Acting Cabotegravir and Rilpivirine Are Highly Effective and Well-Tolerated in People With Human Immunodeficiency Virus Above 65 Years
Source: Open Forum Infect Dis. 2026 Jan 6;13(1):ofaf817. doi: 10.1093/ofid/ofaf817 (PMC12810048; doi:10.1093/ofid/ofaf817)
Supplement: ofaf817_Supplementary_Data [file ofaf817_supplementary_data.docx]

|  | N or median | Missing values | Percentage or IQR |
| --- | --- | --- | --- |
| Age (years) | 67.8 | 0 | 65.0-71.0 |
| Male sex at birth | 116 | 0 | 85.9% |
| BMI (Kg/m^2^) | 25.3 | 9 | 23.5-28.1 |
| BMI≥30 Kg/m^2^ | 19 | 9 | 15% |
| Years living with HIV | 21.0 | 1 | 15.0-28.2 |
| Years on antiretroviral treatment | 19.7 | 5 | 13.0-25.0 |
| HIV RNA  Not detectable  <50 copies/mL  ≥50 copies/mL  Not available (6 months window) | 69  61  3  2 | 2 | 51.1%  45.2%  2.2%  1.5% |
| Baseline antiretroviral treatment  INSTI-based  NNRTI-based  PI-based | 99  28  8 | 0 | 73.3%  20.7%  5.9% |
| HIV subtype A1/A6 | 7 | 18 | 6.0% |
| NNRTI RAMs | 5 | 4 | 3.8% |
| Isolated HBV anti-HBc | 10 | 15 | 8.3% |
| Nadir CD4+ T cell count (n/mm^3^) | 243 | 7 | 116-388 |
| CD4+ T cell count (n/mm^3^) | 607 | 0 | 479-929 |
| Multimorbidity | 41 | 0 | 30.4 |
| Comorbidities  Dyslipidemia  Hypertension  Osteoporosis  Diabetes  Chronic renal disease | 70  43  27  11  8 | 0 | 51.9%  31.9%  20.0%  8.1%  5.9% |
| Polypharmacy | 87 | 0 | 64.4 |

**Supplementary Table 1. Baseline characteristics of the study participants.** “IQR”, interquartile range; “BMI”, body mass index; “NNRTI”, non-nucleoside reverse transcriptase inhibitor; “RAMs”, resistance-associated mutations; “HBV”, Hepatitis B virus; “anti-HBc”, antibodies against HBV core antigen; “INSTI”, integras strand transfer inhibitors; “PI”, protease inhibitors.

| Participant code | Age at BL | Time of LA CAB+RPV interruption  (months) | Reason(s) |
| --- | --- | --- | --- |
| Toxicity or intolerance | | | |
| 3 | 69.8 | 1 | Fever, purulent gluteal abscess |
| 4 | 68.0 | 1 | Palpitations, paresthesias |
| 5 | 65.0 | 1 | Injection site reaction |
| 6 | 72.0 | 3 | Fever, myalgia, skin rash |
| 9 | 74.6 | 7.3 | Injection site reaction, myalgia, fatigue |
| 10 | 70.1 | 10.8 | Fever, myalgia, fatigue |
| 12 | 65.0 | 19.4 | HBV flare |
| Participants’ choice | | | |
| 1 | 69.3 | 1 | Participants’ choice |
| 2 | 65.0 | 1 |  |
| 8 | 73.4 | 6.5 |  |
| 11 | 64.6 | 15.6 |  |
| 13 | 68.7 | 21.3 |  |
| 15 | 65.0 | 57.6 |  |
| Other reasons | | | |
| 7 | 71.0 | 4.6 | Physician’s choice: Presence of NNRTI RAMs |
| 14 | 66.0 | 57.5 | Death |

**Supplementary Table 2. Reasons for treatment interruption in study participants.** “LA CAB+RPV”, Long-acting cabotegravir and rilpivirine; “BL”, baseline; “NNRTI”, non-nucleoside reverse transcriptase inhibitor; “RAMs”, resistance-associated mutations.
